# Supplementary figures and images for: Comparison of shape quantification methods for genomic prediction, and genome-wide association study of sorghum seed morphology
Source: PLoS One. 2019 Nov 21;14(11):e0224695. doi: 10.1371/journal.pone.0224695 (PMC6872133; doi:10.1371/journal.pone.0224695)

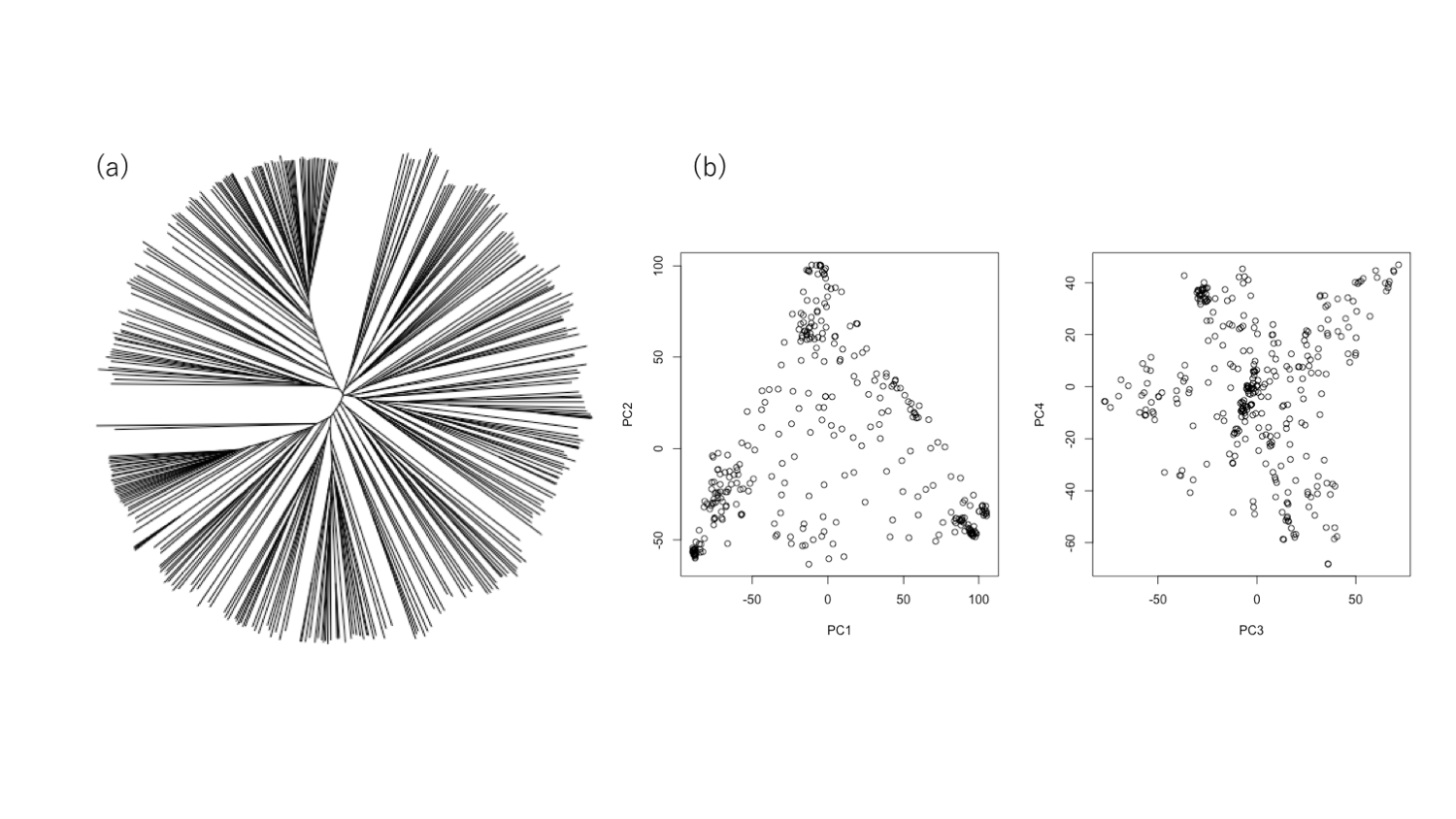

Supplement: S1 Fig — Neighbor-joining trees (a) and PCA plot of these accessions (b). (TIFF) [file pone.0224695.s004.tiff]
