# Supplementary material for: Comparison of shape quantification methods for genomic prediction, and genome-wide association study of sorghum seed morphology
Source: PLoS One. 2019 Nov 21;14(11):e0224695. doi: 10.1371/journal.pone.0224695 (PMC6872133; doi:10.1371/journal.pone.0224695)
Supplement: Attachment — Submitted filename: Response to Reviewers.docx [file pone.0224695.s005.docx]

Response to Reviewers

Reviewer #1

In the abstract, authors highlighted the methods, it would be better to give more background, and while the detailed comparison is important in introduction.

>We added the detailed comparison of geometric morphometrics in the abstract (L25-L28, L36-39).

Is the genome sequencing data (RAD-Seq data) generated in the present study? If yes, I think authors need to provide more detailer results. If not, please cite where the data from. Because I did not find any germplasm information in the MS.

> We added supporting information (S1 Table at L94 and S1 Fig at L112) for providing cultivar names, country names, a neighbor-joining tree and a PCA plot based on the SNP genotypes of these accessions.

In row 158, 168 and 190, for "function ef2cood in shape on R", authors cited two different papers. need to be corrected.

> We corrected them to cite the same paper (the reference #31).

In row 225, authors used rrBLUP to run GWAS, the paper need to be cited. And authors used Q and K in the GWAS, while not mention where the Q and K from.

>We cited a paper related to rrBULP (L263, the refenrence #34), and added methods for calculating Q and K (L240-241).

In the table 1, authors need to show more information, such as p value for the association.

> We added supporting information (S3 Table) for providing p-values of all SNPs analyzed in GWAS.

In row 375, authors wrote "The best procedure for the genetic analysis of biological shape should be chosen based on the shape features", can authors point out which procedure is the best one based on the results of this MS

> Based on the results of this MS, we can mentioned as ”the direction and scaling standardization procedures, however, did affect the results.” (L388). However, because we cannot have a universal definition for “ the best”, it is difficult to choose “the best procedure” even in our case of dataset. To avoid the confusion, we removed the sentence.

Reviewer #2

The authors used ~329 sorghum diverse accessions in this study. However, geo-reference and their race information are not provided in the manuscript. Including this information as a supplemental file will be helpful for future studies with different traits from this population. Also, include a PCA plot of these accessions plotted with the sorghum association panel (Morris et al. 2013. PNAS), and NJ tree depicting their origin and race. These figures will be helpful for sorghum genetics and genomics audience to understand the genetic diversity present in the sorghum germplasm used for this research.

> We added supporting information (S1 Table at L94 and S1 Fig at L112) for providing cultivar names, country names, a neighbor-joining trees and PCA plot based on the SNP genotypes of these accessions.

Recently, sorghum seed mass (with ~1900 geo-referenced accessions) mapped a QTL on chromosome 4 at ~58 Mb (Wang et al., 2019). As findings from Wang et al. (2019) validate the seed shape QTL mapped in this study, cite Wang et al. in the discussion section.

>Thank you for letting us know the paper. We cited Wang et al. (2019) in the introduction and discussion section (L61 and L384).

Minor points: Line 87: change Sorghum × almum to Sorghum x almum

>We corrected Sorghum × almum to Sorghum x almum (L95).
